# Method for Reading Sensors and Controlling Actuators Using Audio Interfaces of Mobile Devices

**DOI:** 10.3390/s120201572

**Published:** 2012-02-06

**Authors:** Rafael V. Aroca, Aquiles F. Burlamaqui, Luiz M. G. Gonçalves

**Affiliations:** NatalNet Laboratory, Technology Center, Computing Engineering and Automation Department, Federal University of Rio Grande do Norte, Natal, RN 59078-900, Brazil; E-Mails: aquiles@natalnet.br (A.F.B.); lmarcos@natalnet.br (L.M.G.G.)

**Keywords:** sensors, smartphones, mobile devices, mechatronics, robotics, robot, control, actuators

## Abstract

This article presents a novel closed loop control architecture based on audio channels of several types of computing devices, such as mobile phones and tablet computers, but not restricted to them. The communication is based on an audio interface that relies on the exchange of audio tones, allowing sensors to be read and actuators to be controlled. As an application example, the presented technique is used to build a low cost mobile robot, but the system can also be used in a variety of mechatronics applications and sensor networks, where smartphones are the basic building blocks.

## 1. Introduction and Motivation

Most robots and automation systems rely on processing units to control their behavior. Such processing units can be embedded processors (such as microcontrollers) or general purpose computers (such as PCs) with specialized Input/Output (I/O) accessories. One common practice is the use of USB devices with several I/O options connected to a PC. Another approach consists of using a microcontroller unit that runs a software algorithm to control the system. Frequently this microcontroller is connected to a computer to monitor and set parameters of the running system.

Although consolidated, these approaches require specific device drivers on the host computer and special configurations. Moreover, if the control algorithm has to be changed, the user must reprogram the microcontroller, which in some cases requires special hardware tools such as in-circuit programmers and debuggers.

Mobile devices such as cell phones, tablets and netbooks are widespread and getting cheaper due to their large production, making them an interesting option to control mechatronics systems. These devices also include several sensors that can be used in a mechatronic system. Examples of sensors available in modern cell phones are the global positioning system receiver (GPS), 3-axis accelerometer, compass, liquid crystal display (LCD) with touchscreen, Internet access via WiFi or GPRS/3G services, camera, speakers, microphone, bluetooth module, light sensor and battery (some have even gyroscope, barometer and stereo camera). If all those accessories would be bought separately and installed in a device, the costs would be more expensive than a device with all these features already included.

To use a mobile device as a mechatronics system main processing unit, a communication link must established between the control unit and the system under control. Some works [1–3] use the mobile’s device RS-232 serial port signals while others use bluetooth [1,4]. In all these works, a microcontroller still needs to be used to communicate with the mobile device, to read sensors and to control actuators. The problem is that not all mobile devices have a serial port, or bluetooth communication interface.

Analyzing modern mobile devices it is possible to note that most of them have an universal communication interface: audio channels, that usually can be accessed through standard 3.5 mm P2 connectors. If there are no connectors for external audio, this article also offers a solution using suction cups to use the proposed system in mobile devices where the speakers or microphones are built-in. Details of such solution are discussed in Section 5.1.

In this article, a system that allows closed loop control using any mobile device that can produce and receive sounds was designed and built. In such system, audio tones are used to encode actuators control commands and sensors states. The system cost is low because only a few parts are needed (these parts are widely used in the telephony industry, making them also easy to find on the market). Moreover the system does not need any intermediate processing unit, making it flexible to easily update the running algorithm, avoiding the need of reprogramming microcontrollers.

### 1.1. Robots and Smartphones

As mentioned, mobile devices have several features that can be used in robotics. Some possibilities of using these features are briefly discussed in the following text. These possibilities are, for sure, non-exhaustive.

- CPU: Modern smartphones already have processors with clock rates of 1 GHz or more. Some models also have multi-core processors. These processing units are capable of doing many complex and computationally intensive operations for autonomous robots navigation. Santos *et al*. [4], for example, showed that it is feasible to execute complex robotics navigation algorithms in processors of older smartphones with 300 MHz clock speed;
- Camera: can be used with a variety of algorithms for visual odometry, object recognition, robot attention (the ability to select a topic of interest [5]), obstacle detection and avoidance, object tracking, and others;
- Compass: can be used to sense the robot’s direction of movement. The system can work with one or two encoders. If only one encoder is used, the compass is used to guarantee that the robot is going in the expected direction and to control the desired curves angles;
- GPS: can be used to obtain the robot position in outdoor environments, altitude and speed;
- Accelerometer: can be used to detect speed changes and consequently if the robot has hit an object in any direction (a virtual bumper). It can also detect the robot’s orientation. It is also possible to use Kalman filtering to do sensor fusion of the camera, encoders and accelerometer to get more accurate positioning;
- Internet: WiFi or other Internet connection can be used to remotely monitor the robot and send commands to it. The robot can also access a cloud system to aid some decision making process and communicate with other robots;
- Bluetooth: can be used to exchange information with nearby robots and for robot localization;
- Bluetooth audio: As the standard audio input and output are used for the control system, a bluetooth headset can be paired with the mobile device, allowing the robot to receive voice commands and give synthesized voice feedback to the user. The Android voice recognizer worked well for both English and Portuguese. The user can press a button in the bluetooth headset and say a complex command such as a phrase. The Android system will then return a vector with most probable phrases that the user has said;
- ROS: The Robot Operating System (ROS) [6] from Willow Garage is already supported in mobile devices running Android using the ros-java branch. Using ROS and the system described in this article, a low cost robot can be built with all the advantages and features of ROS.

### 1.2. Contributions

The main contributions of this work are:

- A novel system for controlling actuators using audio channels
- A novel system for reading sensors information using audio channels
- A closed loop control architecture using the above-mentioned items
- Application of a camera and laser based distance measurement system for robotics
- A low cost mobile robot controlled by smartphones and mobile devices using the techniques introduced in this work

### 1.3. Organization

This paper is structured as follows: Section 2 describes previous architectures for mechatronics systems control. Section 3 introduces the new technique. Section 4 presents the experimental results of the proposed system. Section 5 describes a case study with an application of the system to build a low cost mobile robot and in Section 6 are the final considerations.

## 2. Related Work

This section reviews some of the most relevant related works that uses mobile devices to control robots and their communication interfaces.

### 2.1. Digital Data Interfaces

Santos *et al*. [4] analyze the feasibility of using smartphones to execute robot’s autonomous navigation and localization algorithms. In the proposed system, the robot control algorithm is executed in the mobile phone and the motion commands are sent to the robot using bluetooth. Their experiments are made with mobile phones with processor clocks of 220 MHz and 330 MHz and they conclude that it is possible and robust to execute complex navigation in these devices even with soft real-time requirements. The tested algorithms are well-known: potential fields, particle filter and extended Kalman filter.

Another example of the use of smartphones to control robots is the open source project Cellbots [1] which uses Android based phones to control mobile robots. The project requires a microcontroller that communicates with the phone via bluetooth or serial port and sends the electrical control signals to the motors. The problem is that not all mobile devices have bluetooth or serial ports. Moreover, in some cases the device has the serial port available only internally, requiring disassembly of the device to access the serial port signals. When using bluetooth, the costs are higher because an additional bluetooth module must be installed and connected to the microcontroller.

The work of Hess and Rohrig [7] consists of using mobile phones to remotely control a robot. Their system can connect to the robot using TCP/IP interfaces or bluetooth. In the case of the TCP/IP sockets, the connection to the robot is made using an already existing wireless LAN (WiFi) infrastructure.

Park *et al*. [8] describe user interface techniques for using PDAs or smartphones to remotely control robots. Again, the original robot controller is maintained and the mobile device is used simply as a remote control device. Their system commands are exchanged using WiFi wireless networks.

### 2.2. Analog Audio Interfaces

One interesting alternative is using a dedicated circuit to transform the audio output of the mobile device in a serial port signal [9], but the problem with such approach is that only unidirectional communication is possible, and still, as in the other cases, a microcontroller is needed to decode the serial signal and execute some action.

On the other hand, the telecommunications industry frequently uses the Dial Tone Multi Frequency (DTMF) system to exchange remote control commands between equipments. Section 3.1 contains a description of the DTMF system. The system is more known in telephony for sending the digits that a caller wants to dial to a switching office. DTMF usage to control robots is not new. There are some recent projects that use DTMF digits exchange to remotely control robots: Patil and Henry [10] used a remote mobile phone to telecommand a robot. DTMF tones are sent from the mobile phone to the remote robot’s phone and decoded by a specific integrated circuit and the binary output is connected to a FPGA that controls the robot. Manikandan *et al.* [11] proposed and built a robot that uses two cell phones. One phone is placed in the robot and another acts as a remote control. The DTMF audio produced by the keys pressed in the remote control phone is sent to the phone installed in the robot, and the audio output of this phone is connected to a DTMF decoder via the earphone output of the cell phone. The 4-bit DTMF output is then connected to a microcontroller that interprets the codes and executes the movements related to the keys pressed in the remote control phone. Sai and Sivaramakrishnan [12] used the same setup, where two mobile phones are used, one located at the robot and another used as a remote control. The difference is that the system is applied to different type of robot (mechanically). Naskar *et al.* [13] presented a work where a remote DTMF keypad is used to control a military robot. Some DTMF digits are even used to fire real guns. The main difference is that instead of transmitting the DTMF tones using a phone call, the tones are transmitted using a radio frequency link.

Still about DTMF based control, Ladwa *et al.* [14] proposed a system that can remotely control home appliances or robots via DTMF tones over telephone calls. The tones are generated by keys pressed in a remote phone keypad, received by a phone installed in the system under control and decoded by a DTMF decoder circuit. A microcontroller then executes some pre-programmed action. A similar work is presented by Cho and Jeon [15] where key presses in a remote phone are sent through a telephone call to a receiver cell phone modem. Its audio output is connected to a DTMF decoder chip which is then connected to a robot control board. As the user presses keys in the remote phone, the robot goes forward, backwards or do curves according with the pressed key (a numerical digit that is represented by a DTMF code).

Recently, a startup company created a mobile robot that can be controlled by audio tones from mobile phones [16]. The system is limited to controlling two motors and does not have any feedback or sensor reading capability. The alternative is using camera algorithms such as optical flow to implement visual odometry, but even if so, such limitations make it difficult to build a complete mobile robot because of the lack of important sensory information such as bumpers and distance to objects. Also, the information provided in the company website does not make it clear if the wheel speed can be controlled and which audio frequencies are used.

With the exception of the example in the last paragraph, all other mentioned examples and projects use DTMF tones to remotely control a distant robot over radio or telephone lines. This system, in contrast, uses DTMF tones to control actuators and read sensors in a scheme where the control unit is physically attached to the robot, or near the robot (connected by an audio cable). The advantage is that DTMF tones are very robust to interference and widely adopted, making it easy to find electrical components and software support for dealing with such system.

## 3. System Architecture

This section describes the proposed system architecture. Its main advantage is to provide a universal connection system to read sensors and control actuators of mechatronics systems. The data is exchanged using audio tones, allowing the technique to be used with any device that has audio input/output interfaces.

### 3.1. Theoretical Background

The DTMF system was created in the decade of 1950 as a faster option to the (now obsolete) pulse dialing system. Its main purpose at that time was to send the digits that a caller wants to dial to the switching system of the telephone company. Although almost 60 years old, DTMF is still widely used in telecommunication systems and is still used in most new telephone designs [17].

DTMF tones can be easily heard by pressing the keys on a phone during a telephone call. The system is composed of 16 different audio frequencies organized in a 4 × 4 matrix. Table 1 shows these frequencies and the corresponding keys/digits. A valid digit is always composed by a pair of frequencies (one from the table columns and one from the table rows) transmitted simultaneously. For example, to transmit the digit 9, an audio signal containing the frequencies 852 Hz and 1,477 Hz would have to be generated.

As DTMF was designed to exchange data via telephone lines that can be noisy, the use of 2 frequencies to uniquely identify a digit makes the system efficient and very robust to noise and other sounds that do not characterize a valid DTMF digit. In fact, when the DTMF system was designed, the frequencies were chosen to minimize tone pairs from natural sounds [18].

The described robustness of DTMF led its use in a variety of current remote automation systems such as residential alarm monitoring, vehicle tracking systems and interactive voice response systems such as bank’s automatic answering machines menus that allows the user to execute interactive operations like “Press 1 to check your account balance; Press 2 to block your credit card”.

The wide adoption and reliability of the DTMF system led the semiconductor industry to develop low cost integrated circuits (ICs) that can encode and decode DTMF signals from and to digital binary digits. The system proposed in this article uses such ICs to transmit and receive information. Both actuators control and sensors data are encoded using DTMF tones. The following sections describe the system design.

### 3.2. Device Control

**Figure 1.**
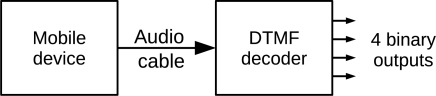
DTMF decoder with four independent outputs controlled by audio from a mobile device.

To control actuators, a mobile device generates a DTMF tone. The tone is decoded by a commercial DTMF decoder chip (such as the MT8870), converting the tone to a 4-bit binary word equivalent to the DTMF input. The decoded output remains present while the DTMF tone is present at the input. The resulting bits can feed a power circuit to control up to four independent binary (on/off) devices such as robots brakes, lights or a pneumatic gripper. Figure 1 shows the basic concept of the system.

The audio output from the mobile device can be directly connected to the input of the DTMF decoder, but in some specific cases an audio preamplifier should be used to enhance the audio amplitude.

**Figure 2.**
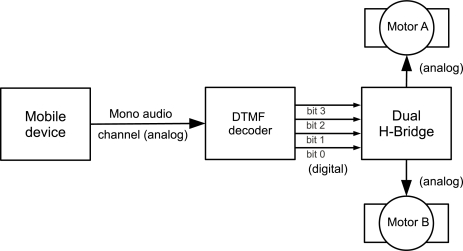
Dual motor control using one mono audio channel.

Figure 2 shows a direct current (DC) motor control application where the 4-bit output of the decoder is connected to a motor control circuit (an H-bridge, for example, using the L298 commercial dual H-bridge IC). As 2 bits are required to control each motor, the system can control 2 DC motors independently. Table 2 shows the DTMF digits and corresponding motor states. Note that these states can be different according to the pin connections between the DTMF decoder and the H-bridge. In order to control the DC motor’s speed, the mobile device turns the DTMF signals on and off in a fixed frequency, mimicking a pulse width modulation (PWM) signal.

To control more devices it is possible to take advantage of the fact that most audio outputs of mobile devices are stereo. Thus, generating different audio tones in the left and right channels doubles the number of controlled devices (8 different on/off devices or 4 DC motors). One interesting option, possible only in devices with USB Host feature, such as netbooks and desktop computers, is to add low cost USB multimedia sound devices, increasing the number of audio ports in the system.

Another possibility consists in directly connecting servo-motors (the ones used in model airplanes) control signals to the output of the DTMF decoder. As each servo needs only one PWM input signal, each stereo audio channel can drive up to eight servo-motors.

### 3.3. Sensor Reading

Most mechatronics systems and sensor networks need sensors to sense the surrounding environment and their own state in order to decide what to do next. To accomplish this task in this system, sensors are connected to the input of a DTMF encoder chip (such as the TCM5087). Each time a sensor state changes, the encoder generates a DTMF tone that is captured and analyzed by the mobile device. According to the digits received it is possible to know which sensor generated the tone. More details on how to identify which sensor and its value are provided in Section 5.1.

**Figure 3.**
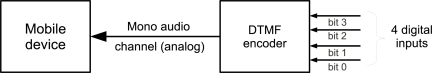
Sensors input using a DTMF generator.

As shown in Figure 3, up to four sensors can be connected to a DTMF encoder that generates tones according to the sensors states. The generator’s output itself is connected to the audio input of the mobile device which continuously samples the audio input checking if the frequency pair that characterizes a DTMF digit is present in the signal. To accomplish this task, the discrete Fourier transform (DFT) is used, according to Equation (1).

(1) $$X(m)=\sum_{n=0}^{N-1}x(n)e^{-j2\pi \mathrm{nm}/N}$$

where X(m) is the frequency magnitude of the signal under analysis at index m, x(n) is the input sequence in time (representing the signal) with n index and N is the DFT number of points. N determines the resolution of the DFT and the number of samples to be analyzed. For performance reasons, a Fast Fourier Transform (FFT) [19] is used to identify the frequency components of the input signal and consequently detect the DTMF digit generated by the DTMF generator that encodes sensor data. For clear and detailed information about these digital signal processing concepts, please refer to Lyons’ book [20] on Digital Signal Processing.

To optimize the FFT computation it is necessary to specify adequate values for N and Fs (the sample rate of the input signal). From Table 1, the highest frequency present in a DTMF tone is 1,633 Hz. Applying the fundamental sampling theorem results in Fs = 3,266 Hz (the theorem states that the sample rate should be at least twice the highest signal to be captured). For implementation convenience and better compatibility, a 8 KHz sample rate is used, which most mobile devices can perform.

The lower the number of points in the FFT, the faster the FFT is computed, and more digits per second can be recognized, leading to a better sensor reading frequency. To compute the smallest adequate number of points for the FFT, Equation (2) is used.

(2) $$f(m)=\frac{\mathrm{mFs}}{N}$$

In Equation (2), f(m) is each frequency under analysis, Fs is the sampling frequency (8 KHz) and N the FFT’s number of points to be minimized. Using N = 256, results in an analysis resolution of about 30 Hz, that is enough to differ from one DTMF frequency component to another. This is consistent with the DFT parameters used by Chitode to detect DTMF digits [21]. The work developed by Khan also uses N = 256 and Fs = 8 KHz to detect DTMF tones [22].

Later in this text, Section 5.1 and Table 3 explains the use and application of this technique to read four digital (on/off) sensors simultaneously.

One of the limitations of the described method is that it is restricted to binary (on/off) sensors. As it is shown is Section 5.1, this is enough for many applications, including measuring angles and speeds using incremental optical encoders. In any case, additional electronics could be used to encode analog signals and transmit these signals using the audio interface. As each DTMF digit encodes 4 bits, the transmission of an analog value converted with a 12-bit analog-digital converter would take 3 transmission cycles. It would also be possible to use digital signal multiplexing hardware to encode more information in the same system (with worse performance). The demultiplexing would be done in the mobile device by software.

## 4. Experimental Results

In order to evaluate the proposed system, an Android application was developed using the Android development kit, which is available for free. Experiments were executed in several mobile devices and desktop computers.

For the actuator control subsystem, experiments showed that generating PWM signals by software is possible, but the resulting signal shows variations (a software generated PWM with 1 ms ON time and 1 ms OFF time produces a real signal with 50 ms ON time and 50 ms OFF time). A better option is to use pre-recorded PWM DTMF tones resulting in high reliability PWM of DTMF tones with frequencies greater than 1 KHz. As mobile devices have mature software support for playing pre-recorded audio, the PWM plays smoothly with low processor usage. In these experiments it was also observed that another practical way of doing fine speed adjustments consists in controlling the audio output volume, resulting in proportional speed changes in the motor(s).

**Figure 4.**
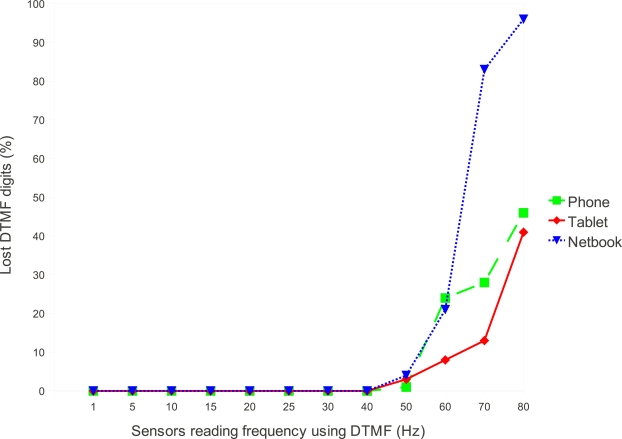
DTMF digit recognition performance in different devices.

Experiments of the sensor reading subsystem are based on the FFT. The experiments showed that in the worst case, the FFT computation time is 17 ms, leading to a theoretical limit of executing up to 58.8 FFTs per second. Figure 4 shows experimental results of the system running on 3 different devices. The tested devices were an early Android based phone, the HTC G1 with a 528 MHz ARM processor, an Android based tablet computer with a dual core 1 GHz ARM processor and a 1 GHz PC netbook with an Intel Celeron processor (in this case a version of the Android operating system for the x86 architecture was used). The FFT was implemented using Java and executed in the virtual machine (dalvik) of the Android system. Using the native development system for Android, thus bypassing the virtual machine, would enhance these results. Another performance improvement can be reached using the Goertzel algorithm [23,24].

From Figure 4 it is possible to note that even the device with less processing power is able to handle about 40 DTMF digits per second with zero packet loss. There are several causes for the increasing packet loss that starts at 40 Hz in the plot. One of the causes are the different audio input timings [25] caused by the different audio hardware of each device. Another cause is related to the task scheduler of the Android operating system (and the underlying Linux kernel) that can be indeterministic when the CPU load is high.

As a reference for comparison, some performance tests were made in a Lego Mindstorms (TM) robotics kit that is commonly used in educational robotics and some scientific researches. When connected to a computer or smartphone via a bluetooth wireless link, the maximum sensor reading rate of the Lego-NXT brick is 20 Hz. If several sensors are used, the bandwidth is divided. For example, using 2 encoders and 2 touch sensors reduces the sensor reading rate to 5 Hz per sensor or less. If the NXT brick is connected to a computer using the USB port, then the maximum sensor reading frequency rises to 166 Hz. If two encoders and two touch sensors (bumpers) are used, then each sensor will be read at a rate of 41.5 Hz. The performance of the system proposed in this article is comparable to this commercial product as a 40 Hz rate can be sustained for each sensor in a system with 4 sensors.

## 5. Case Study Application

The system described in Section 3 can be applied to several situations where a computing device needs to control actuators and read sensors, such as laboratory experiments, machine control and robotics. In this section, a mobile robot case study is described.

### 5.1. Low Cost Mobile Robot

As an application example, the presented technique was used to build a low cost educational mobile robot. For the Robot’s frame, wheels, gears and two motors 24 US dollars were spent. For electronics parts more 6 US dollars were spent summing up a total of 30 US dollars to build the robot. As most people own a mobile phone or a smartphone, there is the assumption that the control device will not have to be bought because a mobile device that the user already has will be used.

Even if the control device needed to be purchased, the option of using a smartphone would still be good because single board computers, typically used in robots or other robot computers, are more expensive than smartphones. Furthermore, smartphones include camera, battery, Internet connection and a variety of sensors that would have to be bought separately and connected to the robot’s computer. With multi-core smartphones running with clock speeds faster than 1 GHz and with 512 MB or 1 GB of RAM memory, they are a good alternative to traditional robots computers.

**Figure 5.**
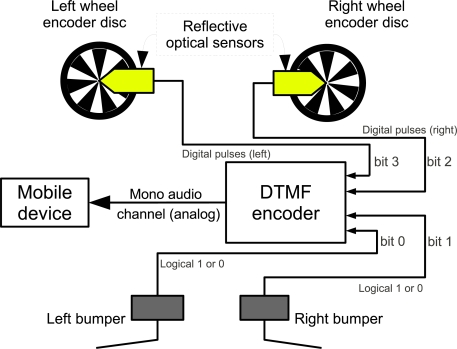
Sensors connection in a mobile robot with differential drive.

Important sensors in such kind of robot are the bumpers to detect collisions and encoders to compute odometry. Figure 5 shows a block diagram connecting bumpers and 2 wheel encoders to the DTMF generator. Table 3 shows a truth table with the possible states of each sensor and the corresponding DTMF digits. Instead of using commercial encoders discs, several encoders were designed and printed with a conventional laser printer. The discs were glued to the robot’s wheels and a standard CNY70 light reflection sensor was mounted in front of each disc.

As can be seen in Table 3, there is a unique DTMF digit that corresponds to each possible sensor state. Using basic binary arithmetic it is possible to obtain the individual state of each sensor. For example, from Table 3 it is known that the bumpers are the bits 0 and 1. Using a bitwise AND operation with the binary mask 0001 will filter all other sensor states and the result will be either 0 or 1, indicating the left bumper state. For the right bumper, the same AND operation can be applied with the binary mask 0010. Furthermore, using the binary 0011 mask and the AND operation will only return a value different than zero if both bumpers are activate at the same time. Using these types of comparisons it is then possible to know the state of each sensor. In the case of the optical encoders, the system’s software monitors for state transitions and add a unit for each transition to a counter that keeps how many pulses each encoder generated.

As seen in the Figure 5, up to four sensors can be connected to each mono audio channel, allowing closed loop control of up to 4 motors if 4 encoders are used. Using the number of pulses accounted for each encoder it is possible to compute displacement and speed for each wheel as it is done with other incremental encoders. This information can be used in classical odometry and localization systems to obtain the robot’s position in a Cartesian space [26,27].

To properly design a robot with the presented technique, a relation between wheel dimensions and the maximum linear speed that can be measured is introduced here. In Equation (3), *V_Max_* is the maximum linear speed of the robot that can be measured, r is radius of the wheel, c is the maximum digits per second detection capacity of the mobile device and s is the encoder disc resolution (number of DTMF digits generated at each complete wheel revolution).

(3) $$V_{\mathrm{Max}}=\frac{2\pi \mathrm{rc}}{s}$$

Table 4 shows the distance measurement resolution and maximum speed that can be measured according to the given equation considering several encoder resolutions.

**Figure 6.**
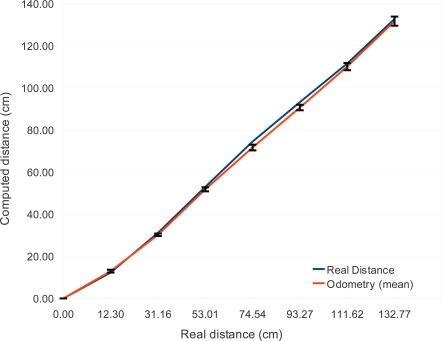
Experimental odometry results. X axis is the real traveled distance manually measured with a tape measure. Y axis is the distance computed by the mobile device using the proposed system with data from the encoders.

Figure 6 shows odometry experimental results for this low cost robot. The error bars are the standard deviation of the real displacement that occurred. The blue line shows the real traveled distance and the red line shows the distance measured by the mobile phone using the proposed technique. Each point in the graph is the average value of ten samples.

According to McComb and Predko, odometry errors are unavoidable due to several factors such as wheels’ slip and small measurement errors in the wheel radius that accumulate over time. They say that a displacements of 6 to 9 meters leads to 15 centimeters odometer error [28] or more, which is a percentual error of 1.6%–2.5%. The greatest odometry error of the system was 3.7% for 74 cm range. But for 130 cm displacements the error was 1 centimeter (0.76%). These values show that the proposed system performance is consistent with classical odometry errors described in the literature.

To close the control loop, the computed odometry information is sent to a classical PI (Proportional-Integral) controller that has as set-points (or goals) the desired distance to be displaced by the robot. The encoders are read at a 40 Hz rate, the position is computed and sent to the controller to decide if the robot has to go faster, stop or walk. If any of the bumpers are activated in the meantime, the control loop is interrupted and the robot immediately stops.

Although most mobile devices have the possibility of recording and reproducing sounds, not all of them have physical connectors for both the audio input and output. To solve this problem in one of the tested devices that does not have an audio input connector, an earphone is attached near the built-in microphone of the device using a suction cup. In this particular case, an audio preamplifier must be used to generate tones with sufficient amplitude to be detected. The DTMF tones encoding sensors data is generated, amplified and sent to the earphone fixed very near the built-in microphone of the mobile device. It is worth mentioning that this scheme works reliably because DTMF system was designed to avoid interference from natural sounds such as music and people’s voices [18].

### 5.2. Human Machine Interface

Users can control this robot from the web or using voice commands. Both a web server and a voice recognition system were implemented. The web-server is embedded into the application, therefore no intermediate computers or servers are needed. Any Internet enabled device can access the web-page and issue several commands to move the robot forward, back, or do curves. For debugging purposes the web-server also shows variable values such as distance, encoder pulses and recognized DTMF pulses from sensors.

The voice recognizer system is straightforward to implement thanks to the Android API. When the user issues a voice command, the operating systems understands it (in several languages) and passes a vector of strings to the robot’s control application with the most probable phrases said. The application just has to select the one that better fits the expected command. Example voice commands are “Walk 30 centimeters” or “Forward 1 meter”. The numbers said by the user are automatically converted to numeric values by the Android API, making it easy to implement softwares that makes the robot move for some distance using closed loop control.

### 5.3. Distance Measurement

An important sensor to aid the navigation of autonomous mobile robots is the distance measurement from the robot to obstacles in front of it. This task is typically performed by ultrasound or laser sensors. Another approach is based on stereo vision, but the computational costs are high. To support distance measurement in this low cost robot, a laser module (laser pointer) is used to project a brilliant red dot in the object in front of the robot. The camera then captures a frame and uses the projected dot position on its image plane to compute the distance to the obstacle based on simple trigonometry. This method is described by Danko [29] and better explained by Portugal-Zambrano and Mena-Chalco [30]. The algorithm assumes that the brightest pixels on the captured image are on the laser projected dot.

**Figure 7.**
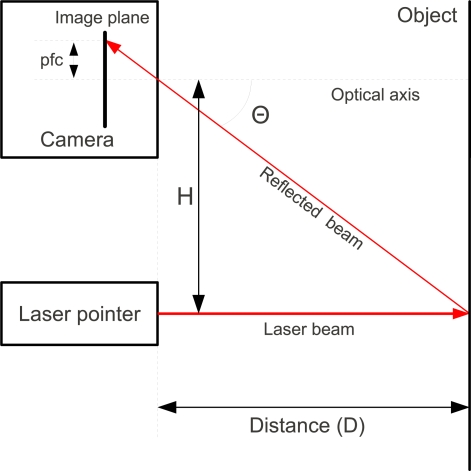
Distance measurement system using a camera and a laser pointer. H is the distance between the camera optical axis and the laser pointer, D the distance between the camera and the object, theta is the angle between the camera’s optical axis and the laser reflected by the object. pfc (pixels from center) is the distance in pixels from the center of the image and the red dot. Figure adapted from Danko and Portugal-Zambrano [29,30].

Figure 7 depicts how the system works. A laser pointer parallel to the camera emits a focused red dot that is projected in an object at distance D from the robot. This red dot is reflected and projected in the camera’s image plane. The distance pfc (pixels from center) between the center of the image plane (in the optical axis) and the red dot in the image plane is proportional to the distance D.

Equation (4) shows how to compute the distance using described system. The distance between the camera and the laser (H) is known previously, the number of pixels from the image center to the red laser dot (pfc) is obtained from the image. The radians per pixels (rpc) and the radian offset (ro) are obtained calibrating the system, which consists of taking several measurements of objects at known distances and their pixels distance from center (pfc). Then a linear regression algorithm finds the best ro and rpc. Details on this calibration can be found on the work of Portugal-Zambrano and Mena-Chalco [30].

(4) $$D=\frac{H}{\tan(\mathrm{pfc}*\mathrm{rpc}+\mathrm{ro}}$$

As can be seem in Equation (4), the measurement range depends mainly on the baseline H given by the distance between the laser and the camera and the number of pixels from the image center, pfc, that has a limit given by the camera resolution. This equation can be used to determine the measurement range. As the object gets farther away, its pfc tends to zero. Assuming pfc to be zero it is possible to simplify Equation (4) to Equation (5) which gives the maximum distance that can be measured. In the same way, the minimum distance is given by half the camera resolution (because the measurement is made from the point to the center of image). Equation (6) specifies the minimum measurement distance. Table 5 shows some possible range values computed using these equations.

(5) $$D_{\max}=\frac{H}{\tan(\mathrm{ro})}$$

(6) $$D_{\min}=\frac{H}{\tan\left(\frac{r}{2}*\mathrm{rpc}+\mathrm{ro}\right)}$$

### 5.4. Block Diagram

**Figure 8.**
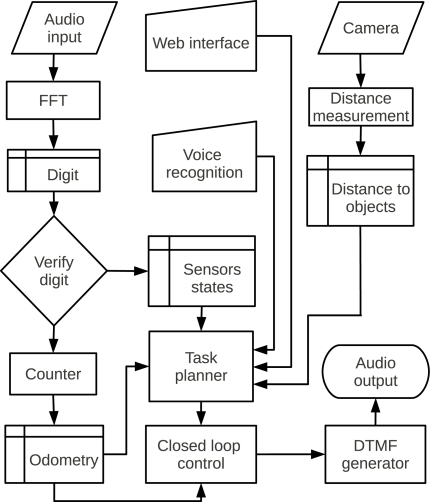
Block diagram of the robot’s software.

Figure 8 shows a block diagram of the system. Each task is executed in a separated thread, thereby reading sensors and controlling motors do not interfere with each other. A task planner module allows the system to integrate the distance measurement, voice commands and web interface. The system running with all these subsystems used between 10% and 45% of the processor in all devices, leaving room to also execute complex algorithms embedded on the robot.

### 5.5. Experimental Results

**Figure 9.**
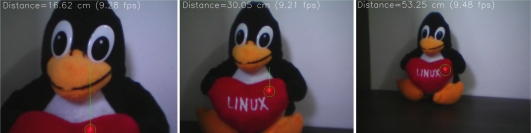
Image seen by the robot’s camera of the same object at different distances. Note the distance of the laser dot to the image center (shown by the green line) when the object is at different distances.

The algorithm implementation is straightforward: the system has to scan a region of the image for a group of pixels with the greatest values (255) in the red channel. The current implementation searches for a pattern of 5 pixels in a cross shape. The center of this cross is the pfc value. Figure 9 shows the results for 3 different distances. The red dot found by the algorithm is shown by a green circle, and the green line shows the distance from the laser dot to the image center.

The baseline used is 4.5 centimeters and after executing a linear regression with a spreadsheet, the calibration values found are ro = 0.074 and rpc = 0.008579.

Table 6 shows experimental results of the system. The average error is 2.55% and the maximum observed error is 8.5%, which happened at the limit of the measurement range. The range of operation goes from 15 cm to 60 cm, but that can be changed modifying the H distance.

The advantage of such system is that the processing needed is very low: the system has to find the brightest red dot on a small limited region of interest in the image, and then compute the distance using simple trigonometric relations. The implementation computes distance at a rate of 9 frames per second in the mobile device while running the FFTs and closed loop control system described. This makes this approach an interesting solution to distance measurement in robotics systems.

**Figure 10.**
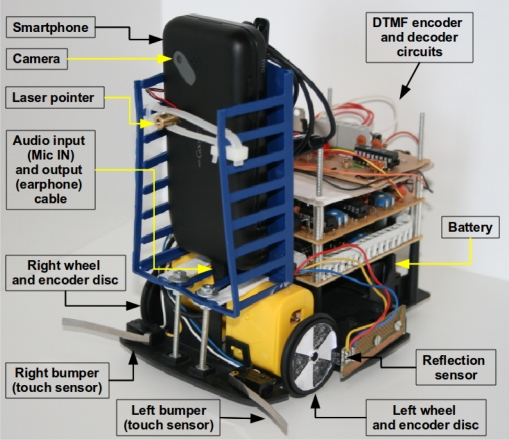
Robot under the control of a mobile phone. The audio input and output channels are connected in a single connector below the phone.

**Figure 11.**
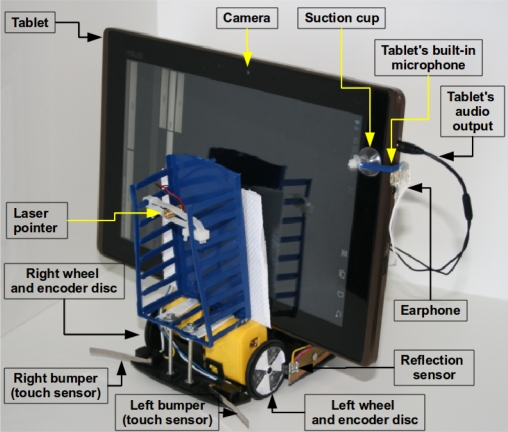
Robot under the control of a tablet computer. The audio output is driven from a P2 connector attached to the earphone jack and the audio input is captured by the built-in microphone of the device. Note the suction cup holding an earphone near the microphone.

**Figure 12.**
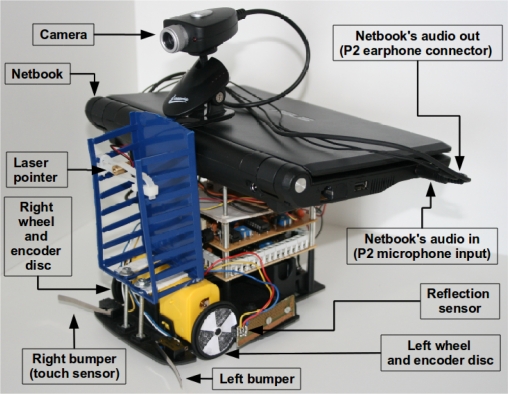
Robot under the control of a netbook computer. Audio input and output channels are connected with independent P2 connectors. This is the most common case for computers.

Figures 10, 11 and 12 show photos of the robot under the control of different devices. Thanks to the portability of the Android system, the same software can be used in PC computers and mobile devices using ARM processors. Although a proof of concept was developed using the Android operating system, the proposed architecture can be used with any system or programming language that can produce and record sounds. One should note that the main contribution of this work is the communication scheme, so these photos show a provisional robot’s assembly setup used to validate the proposed architecture for robotics.

## 6. Conclusions

This paper introduces a simple but universal control architecture that enables a wide variety of devices to implement control of mechatronics and automation systems. The method can be used to implement closed loop control systems in mechatronics systems using audio channels of computing devices, allowing the processing unit to be easily replaced without the need of pairing or special configurations. Several obsolete and current devices can be used to control robots such as PDAs, phones and computers. Even an MP3 player could be used if control without feedback is needed. The sound produced by the player would drive the motors.

As an application example, the presented method is used to build a mobile robot with differential drive. The robot’s complete costs, including frame, motors, sensors and electronics is less than 30 US dollars (in small quantities), and the parts can be easily found in stores or on the Internet. The mentioned price does not include the mobile device.

The method can be used for several applications such as educational robotics, low cost robotics research platforms, telepresence robots, autonomous and remotely controlled robots. In engineering courses it is also a motivation for students to learn digital signal processing theory, and all the other multidisciplinary fields involved in robotics.

Another interesting application of this system is to build sensor networks composed of smartphones that can gather data from their internal sensors and poll external sensors via audio tones, allowing sensor networks to be easily built and scaled using commercial off-the-shelf mobile devices instead of specific boards and development kits.

## Figures and Tables

**Table 1.** DTMF frequencies pairs and corresponding digits. Adapted from the Audio Engineer’s Reference Book [17].

	1,209 Hz	1,336 Hz	1,477 Hz	1,633 Hz
697 Hz	1	2	3	A
770 Hz	4	5	6	B
852 Hz	7	8	9	C
941 Hz	*	0	#	D

**Table 2.** DTMF digits and corresponding motors states.

DTMF digit	Binary word	Motor state	
		Motor A	Motor B
None	00 00	Stopped	Stopped
D	00 00	Stopped	Stopped
1	00 01	Stopped	Left spin
2	00 10	Stopped	Right spin
3	00 11	Stopped	Invalid
4	01 00	Left spin	Stopped
5	01 01	Left spin	Left spin
6	01 10	Left spin	Right spin
7	01 11	Left spin	Invalid
8	10 00	Right spin	Stopped
9	10 01	Right spin	Left spin
0	10 10	Right spin	Right spin
*	10 11	Right spin	Invalid
#	11 00	Invalid	Stopped
A	11 01	Invalid	Left spin
B	11 10	Invalid	Right spin
C	11 11	Invalid	Invalid

**Table 3.** Truth table used with 4 sensors input used in the case study robot.

Left Encoder (bit 3)	Right encoder (bit 2)	Right bumper (bit 1)	Left bumper (bit 0)	DTMF Digit
0	0	0	0	0
0	0	0	1	1
0	0	1	0	2
0	0	1	1	3
0	1	0	0	4
0	1	0	1	5
0	1	1	0	6
0	1	1	1	7
1	0	0	0	8
1	0	0	1	9
1	0	1	0	A
1	0	1	1	B
1	1	0	0	C
1	1	0	1	D
1	1	1	0	*
1	1	1	1	#

**Table 4.** Encoder resolution, displacement measurement resolution and maximum speed that can be measured (considering r = 25 mm and s = 40 DTMF digits per second).

Encoder resolution (digits per revolution)	Displacement resolution	Maximum speed
1	157 mm	6,283 mm/s
6	26 mm	1,047 mm/s
12	13 mm	571 mm/s
24	6.5 mm	261 mm/s
40	3.92 mm	157 mm/s

**Table 5.** Measurement range for a VGA camera (640 × 480). All values in centimeters.

H	Measurement Range	
	Minimum	Maximum
0.5	1.74	6.74
1	3.48	13.48
4.5	15.65	60.68
10	34.79	134.86

**Table 6.** Distance measurement results using a mobile phone camera.

Real distance (cm)	Measured distance (cm)	Error (%)
16	16.02	0.12
20	20.30	1.50
25	25.29	1.16
30	29.70	1.00
35	35.43	1.22
40	39.79	0.50
45	43.80	2.66
50	47.80	4.40
55	52.57	4.41
60	54.90	8.50
